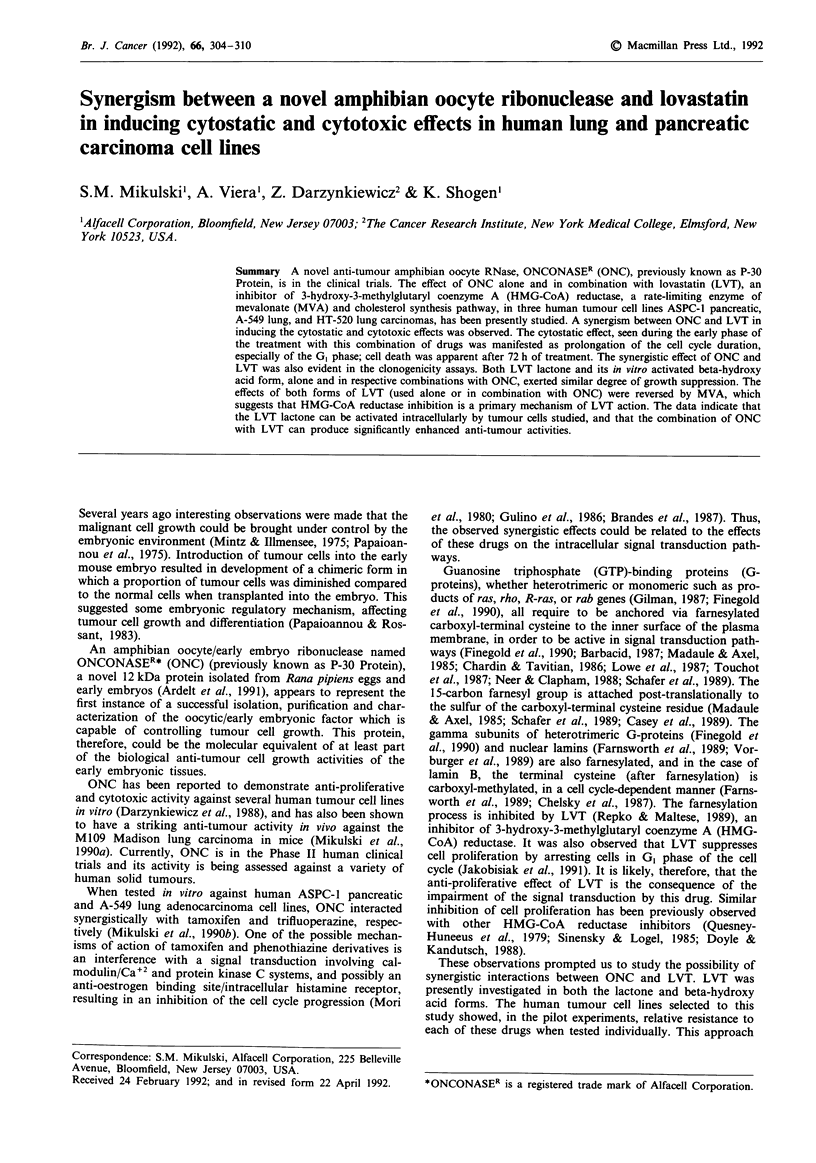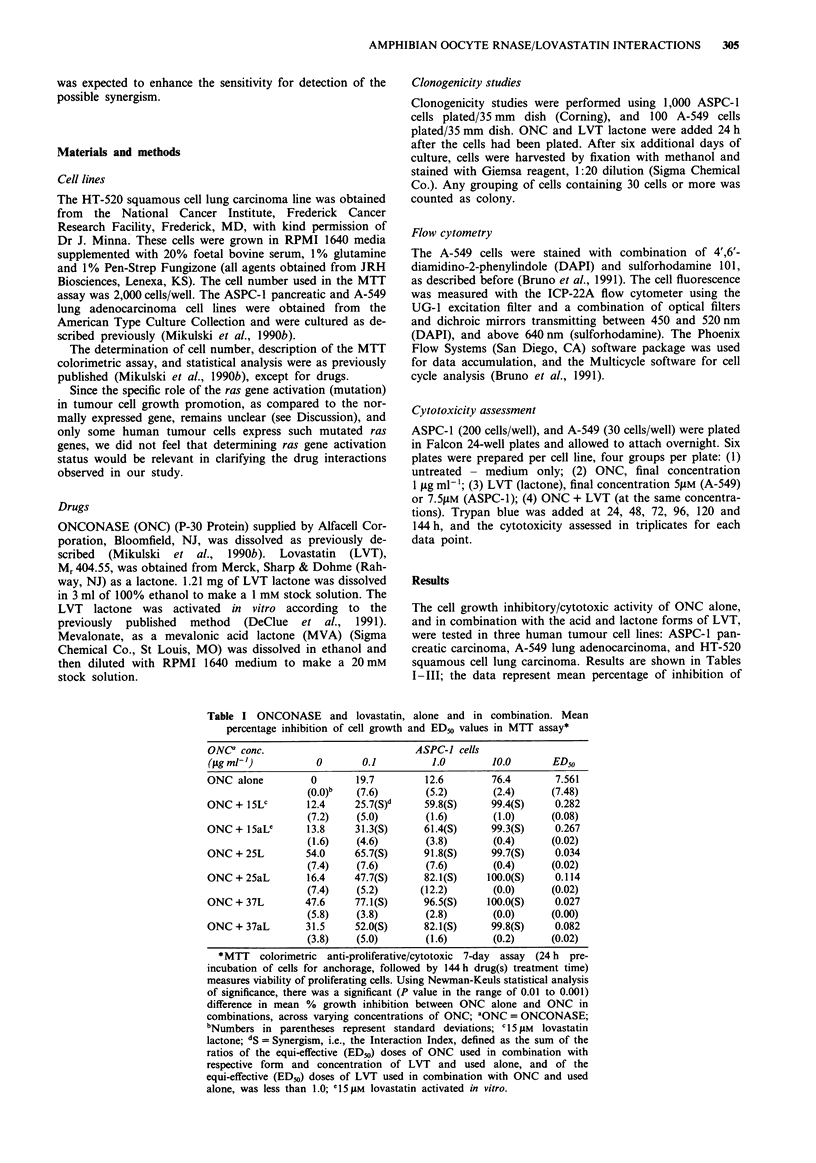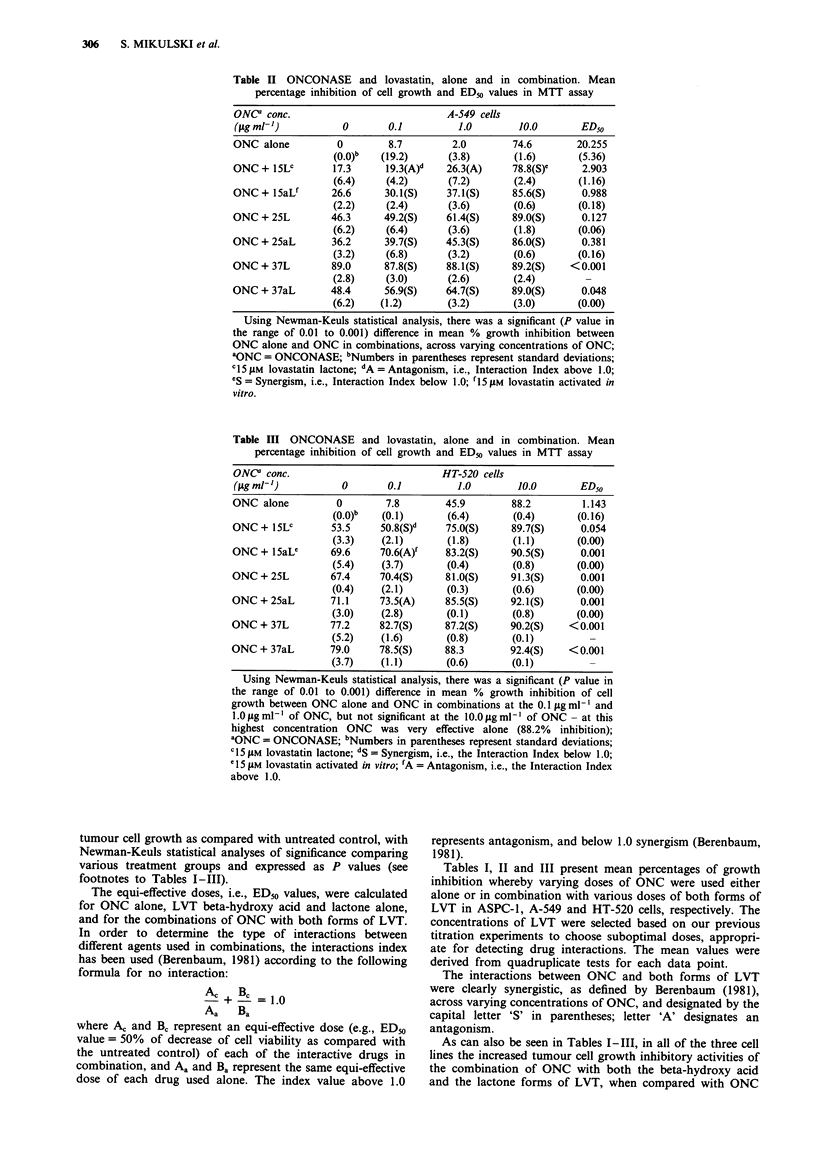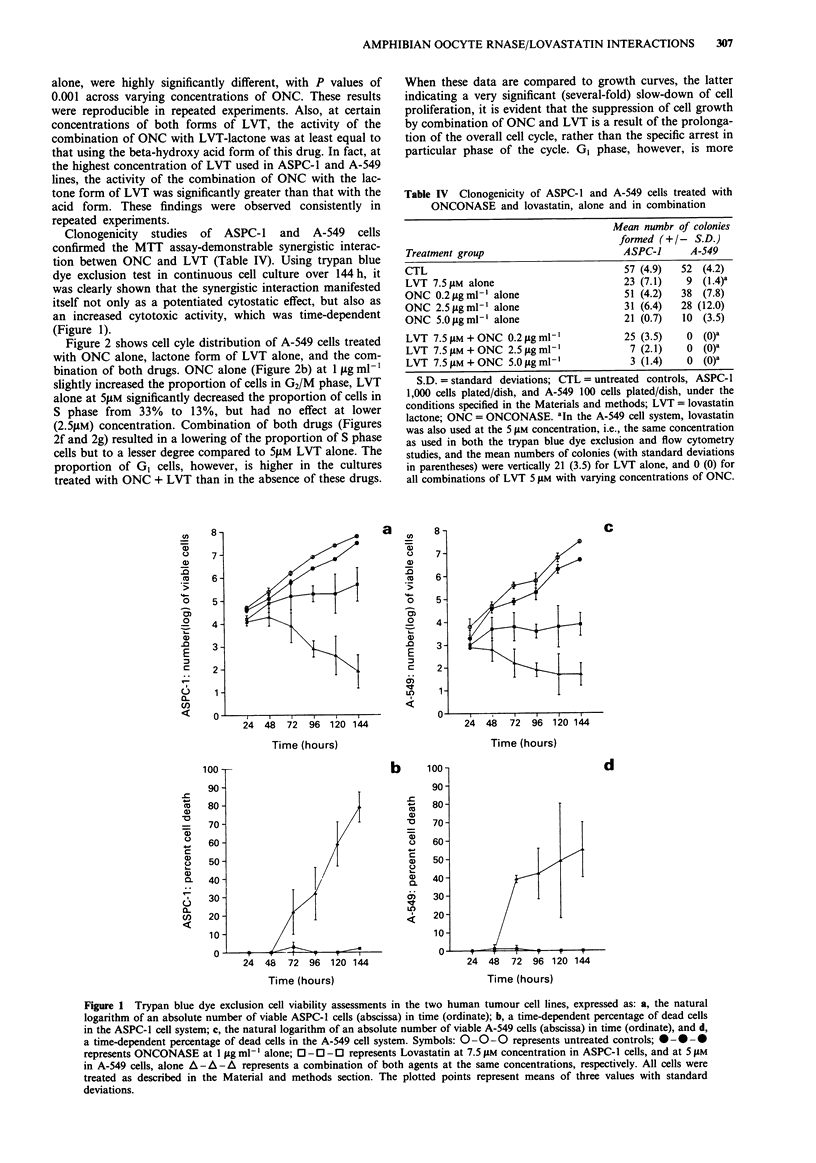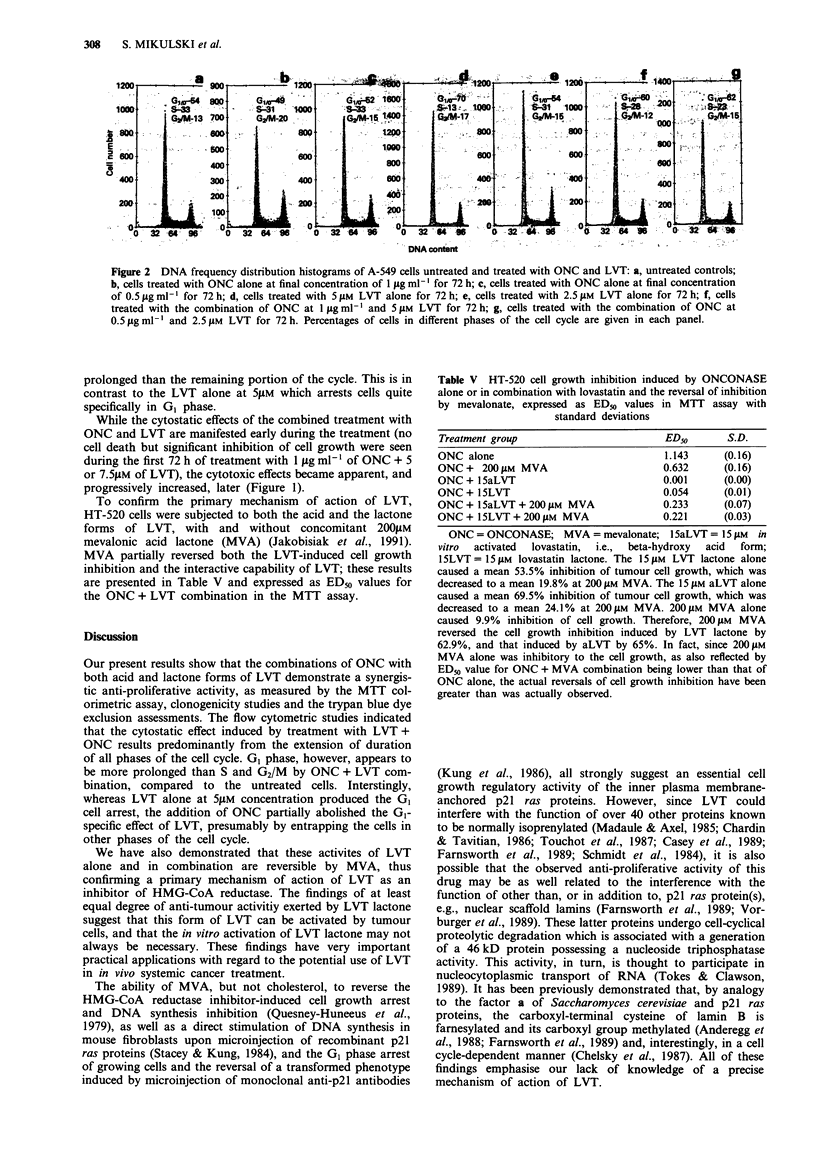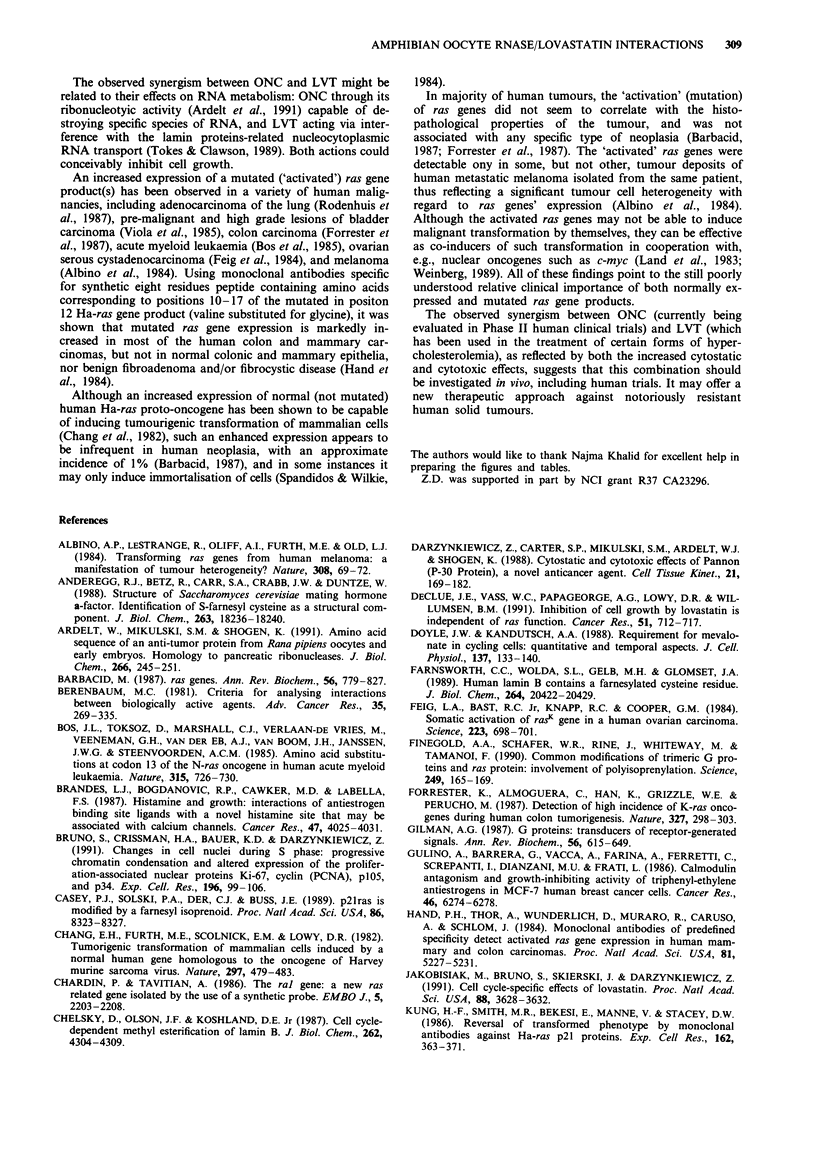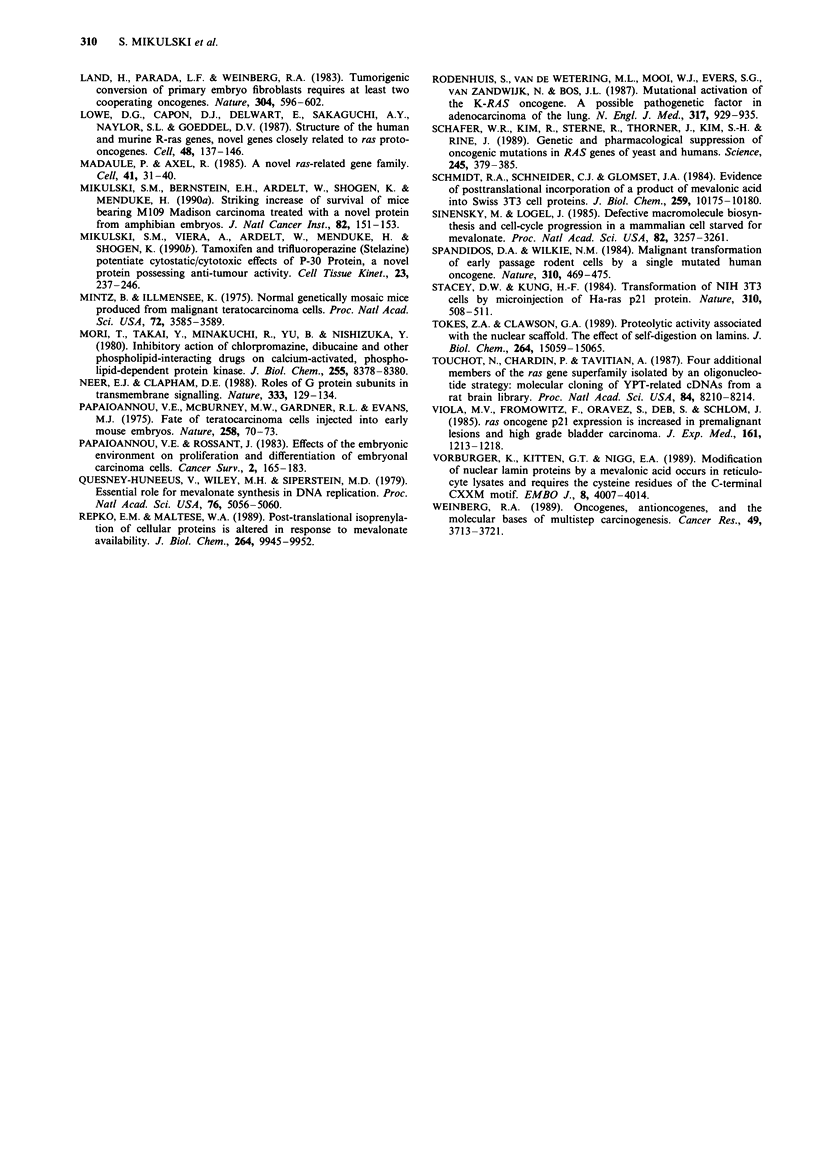# Synergism between a novel amphibian oocyte ribonuclease and lovastatin in inducing cytostatic and cytotoxic effects in human lung and pancreatic carcinoma cell lines.

**DOI:** 10.1038/bjc.1992.261

**Published:** 1992-08

**Authors:** S. M. Mikulski, A. Viera, Z. Darzynkiewicz, K. Shogen

**Affiliations:** Alfacell Corporation, Bloomfield, New Jersey 07003.

## Abstract

A novel anti-tumour amphibian oocyte RNase, ONCONASER (ONC), previously known as P-30 Protein, is in the clinical trials. The effect of ONC alone and in combination with lovastatin (LVT), an inhibitor of 3-hydroxy-3-methylglutaryl coenzyme A (HMG-CoA) reductase, a rate-limiting enzyme of mevalonate (MVA) and cholesterol synthesis pathway, in three human tumour cell lines ASPC-1 pancreatic, A-549 lung, and HT-520 lung carcinomas, has been presently studied. A synergism between ONC and LVT in inducing the cytostatic and cytotoxic effects was observed. The cytostatic effect, seen during the early phase of the treatment with this combination of drugs was manifested as prolongation of the cell cycle duration, especially of the G1 phase; cell death was apparent after 72 h of treatment. The synergistic effect of ONC and LVT was also evident in the clonogenicity assays. Both LVT lactone and its in vitro activated beta-hydroxy acid form, alone and in respective combinations with ONC, exerted similar degree of growth suppression. The effects of both forms of LVT (used alone or in combination with ONC) were reversed by MVA, which suggests that HMG-CoA reductase inhibition is a primary mechanism of LVT action. The data indicate that the LVT lactone can be activated intracellularly by tumour cells studied, and that the combination of ONC with LVT can produce significantly enhanced anti-tumour activities.


Br. J. Cancer (1992), 66, 304–310 © Macmillan Press Ltd., 1992

### Synergism between a novel amphibian oocyte ribonuclease and lovastatin in inducing cytostatic and cytotoxic effects in human lung and pancreatic carcinoma cell lines

S.M. Mikulski[1], A. Viera[1], Z. Darzynkiewicz[2] & K. Shogen[1]

[1]*Alfacell Corporation, Bloomfield, New Jersey 07003;* [2]*The Cancer Research Institute, New York Medical College, Elmsford, New York 10523, USA.*

**Summary** A novel anti-tumour amphibian oocyte RNase, ONCONASE[R] (ONC), previously known as P-30 Protein, is in the clinical trials. The effect of ONC alone and in combination with lovastatin (LVT), an inhibitor of 3-hydroxy-3-methylglutaryl coenzyme A (HMG-CoA) reductase, a rate-limiting enzyme of mevalonate (MVA) and cholesterol synthesis pathway, in three human tumour cell lines ASPC-1 pancreatic, A-549 lung, and HT-520 lung carcinomas, has been presently studied. A synergism between ONC and LVT in inducing the cytostatic and cytotoxic effects was observed. The cytostatic effect, seen during the early phase of the treatment with this combination of drugs was manifested as prolongation of the cell cycle duration, especially of the $G_1$ phase; cell death was apparent after 72 h of treatment. The synergistic effect of ONC and LVT was also evident in the clonogenicity assays. Both LVT lactone and its *in vitro* activated beta-hydroxy acid form, alone and in respective combinations with ONC, exerted similar degree of growth suppression. The effects of both forms of LVT (used alone or in combination with ONC) were reversed by MVA, which suggests that HMG-CoA reductase inhibition is a primary mechanism of LVT action. The data indicate that the LVT lactone can be activated intracellularly by tumour cells studied, and that the combination of ONC with LVT can produce significantly enhanced anti-tumour activities.

Several years ago interesting observations were made that the malignant cell growth could be brought under control by the embryonic environment (Mintz & Illmensee, 1975; Papaioannou *et al.*, 1975). Introduction of tumour cells into the early mouse embryo resulted in development of a chimeric form in which a proportion of tumour cells was diminished compared to the normal cells when transplanted into the embryo. This suggested some embryonic regulatory mechanism, affecting tumour cell growth and differentiation (Papaioannou & Rossant, 1983).

An amphibian oocyte/early embryo ribonuclease named ONCONASE[R]* (ONC) (previously known as P-30 Protein), a novel 12 kDa protein isolated from *Rana pipiens* eggs and early embryos (Ardelt *et al.*, 1991), appears to represent the first instance of a successful isolation, purification and characterization of the oocytic/early embryonic factor which is capable of controlling tumour cell growth. This protein, therefore, could be the molecular equivalent of at least part of the biological anti-tumour cell growth activities of the early embryonic tissues.

ONC has been reported to demonstrate anti-proliferative and cytotoxic activity against several human tumour cell lines *in vitro* (Darzynkiewicz *et al.*, 1988), and has also been shown to have a striking anti-tumour activity *in vivo* against the M109 Madison lung carcinoma in mice (Mikulski *et al.*, 1990*a*). Currently, ONC is in the Phase II human clinical trials and its activity is being assessed against a variety of human solid tumours.

When tested *in vitro* against human ASPC-1 pancreatic and A-549 lung adenocarcinoma cell lines, ONC interacted synergistically with tamoxifen and trifluoperazine, respectively (Mikulski *et al.*, 1990*b*). One of the possible mechanisms of action of tamoxifen and phenothiazine derivatives is an interference with a signal transduction involving calmodulin/$Ca^{+2}$ and protein kinase C systems, and possibly an anti-oestrogen binding site/intracellular histamine receptor, resulting in an inhibition of the cell cycle progression (Mori *et al.*, 1980; Gulino *et al.*, 1986; Brandes *et al.*, 1987). Thus, the observed synergistic effects could be related to the effects of these drugs on the intracellular signal transduction pathways.

Guanosine triphosphate (GTP)-binding proteins (G-proteins), whether heterotrimeric or monomeric such as products of *ras*, *rho*, *R-ras*, or *rab* genes (Gilman, 1987; Finegold *et al.*, 1990), all require to be anchored via farnesylated carboxyl-terminal cysteine to the inner surface of the plasma membrane, in order to be active in signal transduction pathways (Finegold *et al.*, 1990; Barbacid, 1987; Madaule & Axel, 1985; Chardin & Tavitian, 1986; Lowe *et al.*, 1987; Touchot *et al.*, 1987; Neer & Clapham, 1988; Schafer *et al.*, 1989). The 15-carbon farnesyl group is attached post-translationally to the sulfur of the carboxyl-terminal cysteine residue (Madaule & Axel, 1985; Schafer *et al.*, 1989; Casey *et al.*, 1989). The gamma subunits of heterotrimeric G-proteins (Finegold *et al.*, 1990) and nuclear lamins (Farnsworth *et al.*, 1989; Vorburger *et al.*, 1989) are also farnesylated, and in the case of lamin B, the terminal cysteine (after farnesylation) is carboxyl-methylated, in a cell cycle-dependent manner (Farnsworth *et al.*, 1989; Chelsky *et al.*, 1987). The farnesylation process is inhibited by LVT (Repko & Maltese, 1989), an inhibitor of 3-hydroxy-3-methylglutaryl coenzyme A (HMG-CoA) reductase. It was also observed that LVT suppresses cell proliferation by arresting cells in $G_1$ phase of the cell cycle (Jakobisiak *et al.*, 1991). It is likely, therefore, that the anti-proliferative effect of LVT is the consequence of the impairment of the signal transduction by this drug. Similar inhibition of cell proliferation has been previously observed with other HMG-CoA reductase inhibitors (Quesney-Huneeus *et al.*, 1979; Sinensky & Logel, 1985; Doyle & Kandutsch, 1988).

These observations prompted us to study the possibility of synergistic interactions between ONC and LVT. LVT was presently investigated in both the lactone and beta-hydroxy acid forms. The human tumour cell lines selected to this study showed, in the pilot experiments, relative resistance to each of these drugs when tested individually. This approach

Correspondence: S.M. Mikulski, Alfacell Corporation, 225 Belleville Avenue, Bloomfield, New Jersey 07003, USA.
Received 24 February 1992; and in revised form 22 April 1992.

*ONCONASE[R] is a registered trade mark of Alfacell Corporation.

was expected to enhance the sensitivity for detection of the possible synergism.

### Materials and methods

### Cell lines

The HT-520 squamous cell lung carcinoma line was obtained from the National Cancer Institute, Frederick Cancer Research Facility, Frederick, MD, with kind permission of Dr J. Minna. These cells were grown in RPMI 1640 media supplemented with 20% foetal bovine serum, 1% glutamine and 1% Pen-Strep Fungizone (all agents obtained from JRH Biosciences, Lenexa, KS). The cell number used in the MTT assay was 2,000 cells/well. The ASPC-1 pancreatic and A-549 lung adenocarcinoma cell lines were obtained from the American Type Culture Collection and were cultured as described previously (Mikulski *et al.*, 1990*b*).

The determination of cell number, description of the MTT colorimetric assay, and statistical analysis were as previously published (Mikulski *et al.*, 1990*b*), except for drugs.

Since the specific role of the *ras* gene activation (mutation) in tumour cell growth promotion, as compared to the normally expressed gene, remains unclear (see Discussion), and only some human tumour cells express such mutated *ras* genes, we did not feel that determining *ras* gene activation status would be relevant in clarifying the drug interactions observed in our study.

### Drugs

ONCONASE (ONC) (P-30 Protein) supplied by Alfacell Corporation, Bloomfield, NJ, was dissolved as previously described (Mikulski *et al.*, 1990*b*). Lovastatin (LVT), $M_r$ 404.55, was obtained from Merck, Sharp & Dohme (Rahway, NJ) as a lactone. 1.21 mg of LVT lactone was dissolved in 3 ml of 100% ethanol to make a 1 mM stock solution. The LVT lactone was activated *in vitro* according to the previously published method (DeClue *et al.*, 1991). Mevalonate, as a mevalonic acid lactone (MVA) (Sigma Chemical Co., St Louis, MO) was dissolved in ethanol and then diluted with RPMI 1640 medium to make a 20 mM stock solution.

### Clonogenicity studies

Clonogenicity studies were performed using 1,000 ASPC-1 cells plated/35 mm dish (Corning), and 100 A-549 cells plated/35 mm dish. ONC and LVT lactone were added 24 h after the cells had been plated. After six additional days of culture, cells were harvested by fixation with methanol and stained with Giemsa reagent, 1:20 dilution (Sigma Chemical Co.). Any grouping of cells containing 30 cells or more was counted as colony.

### Flow cytometry

The A-549 cells were stained with combination of 4′,6′-diamidino-2-phenylindole (DAPI) and sulforhodamine 101, as described before (Bruno *et al.*, 1991). The cell fluorescence was measured with the ICP-22A flow cytometer using the UG-1 excitation filter and a combination of optical filters and dichroic mirrors transmitting between 450 and 520 nm (DAPI), and above 640 nm (sulforhodamine). The Phoenix Flow Systems (San Diego, CA) software package was used for data accumulation, and the Multicycle software for cell cycle analysis (Bruno *et al.*, 1991).

### Cytotoxicity assessment

ASPC-1 (200 cells/well), and A-549 (30 cells/well) were plated in Falcon 24-well plates and allowed to attach overnight. Six plates were prepared per cell line, four groups per plate: (1) untreated – medium only; (2) ONC, final concentration 1 μg $ml^{-1}$; (3) LVT (lactone), final concentration 5μM (A-549) or 7.5μM (ASPC-1); (4) ONC + LVT (at the same concentrations). Trypan blue was added at 24, 48, 72, 96, 120 and 144 h, and the cytotoxicity assessed in triplicates for each data point.

### Results

The cell growth inhibitory/cytotoxic activity of ONC alone, and in combination with the acid and lactone forms of LVT, were tested in three human tumour cell lines: ASPC-1 pancreatic carcinoma, A-549 lung adenocarcinoma, and HT-520 squamous cell lung carcinoma. Results are shown in Tables I–III; the data represent mean percentage of inhibition of

**Table I** ONCONASE and lovastatin, alone and in combination. Mean percentage inhibition of cell growth and $ED_{50}$ values in MTT assay*

| *ONC[a] conc.* | | | *ASPC-1 cells* | | |
|---|---|---|---|---|---|
| *(μg $ml^{-1}$)* | *0* | *0.1* | *1.0* | *10.0* | *$ED_{50}$* |
| ONC alone | 0 | 19.7 | 12.6 | 76.4 | 7.561 |
| | (0.0)[b] | (7.6) | (5.2) | (2.4) | (7.48) |
| ONC + 15L[c] | 12.4 | 25.7(S)[d] | 59.8(S) | 99.4(S) | 0.282 |
| | (7.2) | (5.0) | (1.6) | (1.0) | (0.08) |
| ONC + 15aL[e] | 13.8 | 31.3(S) | 61.4(S) | 99.3(S) | 0.267 |
| | (1.6) | (4.6) | (3.8) | (0.4) | (0.02) |
| ONC + 25L | 54.0 | 65.7(S) | 91.8(S) | 99.7(S) | 0.034 |
| | (7.4) | (7.6) | (7.6) | (0.4) | (0.02) |
| ONC + 25aL | 16.4 | 47.7(S) | 82.1(S) | 100.0(S) | 0.114 |
| | (7.4) | (5.2) | (12.2) | (0.0) | (0.02) |
| ONC + 37L | 47.6 | 77.1(S) | 96.5(S) | 100.0(S) | 0.027 |
| | (5.8) | (3.8) | (2.8) | (0.0) | (0.00) |
| ONC + 37aL | 31.5 | 52.0(S) | 82.1(S) | 99.8(S) | 0.082 |
| | (3.8) | (5.0) | (1.6) | (0.2) | (0.02) |

*MTT colorimetric anti-proliferative/cytotoxic 7-day assay (24 h pre-incubation of cells for anchorage, followed by 144 h drug(s) treatment time) measures viability of proliferating cells. Using Newman-Keuls statistical analysis of significance, there was a significant (*P* value in the range of 0.01 to 0.001) difference in mean % growth inhibition between ONC alone and ONC in combinations, across varying concentrations of ONC; [a]ONC = ONCONASE; [b]Numbers in parentheses represent standard deviations; [c]15 μM lovastatin lactone; [d]S = Synergism, i.e., the Interaction Index, defined as the sum of the ratios of the equi-effective ($ED_{50}$) doses of ONC used in combination with respective form and concentration of LVT and used alone, and of the equi-effective ($ED_{50}$) doses of LVT used in combination with ONC and used alone, was less than 1.0; [e]15 μM lovastatin activated *in vitro*.

**Table II** ONCONASE and lovastatin, alone and in combination. Mean percentage inhibition of cell growth and $ED_{50}$ values in MTT assay

| *ONC[a] conc.* | *A-549 cells* | | | | |
|---|---|---|---|---|---|
| *(μg ml⁻¹)* | *0* | *0.1* | *1.0* | *10.0* | *$ED_{50}$* |
| ONC alone | 0 | 8.7 | 2.0 | 74.6 | 20.255 |
| | (0.0)[b] | (19.2) | (3.8) | (1.6) | (5.36) |
| ONC + 15L[c] | 17.3 | 19.3(A)[d] | 26.3(A) | 78.8(S)[e] | 2.903 |
| | (6.4) | (4.2) | (7.2) | (2.4) | (1.16) |
| ONC + 15aL[f] | 26.6 | 30.1(S) | 37.1(S) | 85.6(S) | 0.988 |
| | (2.2) | (2.4) | (3.6) | (0.6) | (0.18) |
| ONC + 25L | 46.3 | 49.2(S) | 61.4(S) | 89.0(S) | 0.127 |
| | (6.2) | (6.4) | (3.6) | (1.8) | (0.06) |
| ONC + 25aL | 36.2 | 39.7(S) | 45.3(S) | 86.0(S) | 0.381 |
| | (3.2) | (6.8) | (3.2) | (0.6) | (0.16) |
| ONC + 37L | 89.0 | 87.8(S) | 88.1(S) | 89.2(S) | <0.001 |
| | (2.8) | (3.0) | (2.6) | (2.4) | – |
| ONC + 37aL | 48.4 | 56.9(S) | 64.7(S) | 89.0(S) | 0.048 |
| | (6.2) | (1.2) | (3.2) | (3.0) | (0.00) |

Using Newman-Keuls statistical analysis, there was a significant (*P* value in the range of 0.01 to 0.001) difference in mean % growth inhibition between ONC alone and ONC in combinations, across varying concentrations of ONC; [a]ONC = ONCONASE; [b]Numbers in parentheses represent standard deviations; [c]15 μM lovastatin lactone; [d]A = Antagonism, i.e., Interaction Index above 1.0; [e]S = Synergism, i.e., Interaction Index below 1.0; [f]15 μM lovastatin activated *in vitro*.

**Table III** ONCONASE and lovastatin, alone and in combination. Mean percentage inhibition of cell growth and $ED_{50}$ values in MTT assay

| *ONC[a] conc.* | *HT-520 cells* | | | | |
|---|---|---|---|---|---|
| *(μg ml⁻¹)* | *0* | *0.1* | *1.0* | *10.0* | *$ED_{50}$* |
| ONC alone | 0 | 7.8 | 45.9 | 88.2 | 1.143 |
| | (0.0)[b] | (0.1) | (6.4) | (0.4) | (0.16) |
| ONC + 15L[c] | 53.5 | 50.8(S)[d] | 75.0(S) | 89.7(S) | 0.054 |
| | (3.3) | (2.1) | (1.8) | (1.1) | (0.00) |
| ONC + 15aL[e] | 69.6 | 70.6(A)[f] | 83.2(S) | 90.5(S) | 0.001 |
| | (5.4) | (3.7) | (0.4) | (0.8) | (0.00) |
| ONC + 25L | 67.4 | 70.4(S) | 81.0(S) | 91.3(S) | 0.001 |
| | (0.4) | (2.1) | (0.3) | (0.6) | (0.00) |
| ONC + 25aL | 71.1 | 73.5(A) | 85.5(S) | 92.1(S) | 0.001 |
| | (3.0) | (2.8) | (0.1) | (0.8) | (0.00) |
| ONC + 37L | 77.2 | 82.7(S) | 87.2(S) | 90.2(S) | <0.001 |
| | (5.2) | (1.6) | (0.8) | (0.1) | – |
| ONC + 37aL | 79.0 | 78.5(S) | 88.3 | 92.4(S) | <0.001 |
| | (3.7) | (1.1) | (0.6) | (0.1) | – |

Using Newman-Keuls statistical analysis, there was a significant (*P* value in the range of 0.01 to 0.001) difference in mean % growth inhibition of cell growth between ONC alone and ONC in combinations at the 0.1 μg ml⁻¹ and 1.0 μg ml⁻¹ of ONC, but not significant at the 10.0 μg ml⁻¹ of ONC – at this highest concentration ONC was very effective alone (88.2% inhibition); [a]ONC = ONCONASE; [b]Numbers in parentheses represent standard deviations; [c]15 μM lovastatin lactone; [d]S = Synergism, i.e., the Interaction Index below 1.0; [e]15 μM lovastatin activated *in vitro*; [f]A = Antagonism, i.e., the Interaction Index above 1.0.

tumour cell growth as compared with untreated control, with Newman-Keuls statistical analyses of significance comparing various treatment groups and expressed as *P* values (see footnotes to Tables I–III).

The equi-effective doses, i.e., $ED_{50}$ values, were calculated for ONC alone, LVT beta-hydroxy acid and lactone alone, and for the combinations of ONC with both forms of LVT. In order to determine the type of interactions between different agents used in combinations, the interactions index has been used (Berenbaum, 1981) according to the following formula for no interaction:

$$\frac{A_c}{A_a} + \frac{B_c}{B_a} = 1.0$$

where $A_c$ and $B_c$ represent an equi-effective dose (e.g., $ED_{50}$ value = 50% of decrease of cell viability as compared with the untreated control) of each of the interactive drugs in combination, and $A_a$ and $B_a$ represent the same equi-effective dose of each drug used alone. The index value above 1.0 represents antagonism, and below 1.0 synergism (Berenbaum, 1981).

Tables I, II and III present mean percentages of growth inhibition whereby varying doses of ONC were used either alone or in combination with various doses of both forms of LVT in ASPC-1, A-549 and HT-520 cells, respectively. The concentrations of LVT were selected based on our previous titration experiments to choose suboptimal doses, appropriate for detecting drug interactions. The mean values were derived from quadruplicate tests for each data point.

The interactions between ONC and both forms of LVT were clearly synergistic, as defined by Berenbaum (1981), across varying concentrations of ONC, and designated by the capital letter 'S' in parentheses; letter 'A' designates an antagonism.

As can also be seen in Tables I–III, in all of the three cell lines the increased tumour cell growth inhibitory activities of the combination of ONC with both the beta-hydroxy acid and the lactone forms of LVT, when compared with ONC

alone, were highly significantly different, with $P$ values of 0.001 across varying concentrations of ONC. These results were reproducible in repeated experiments. Also, at certain concentrations of both forms of LVT, the activity of the combination of ONC with LVT-lactone was at least equal to that using the beta-hydroxy acid form of this drug. In fact, at the highest concentration of LVT used in ASPC-1 and A-549 lines, the activity of the combination of ONC with the lactone form of LVT was significantly greater than that with the acid form. These findings were observed consistently in repeated experiments.

Clonogenicity studies of ASPC-1 and A-549 cells confirmed the MTT assay-demonstrable synergistic interaction betwen ONC and LVT (Table IV). Using trypan blue dye exclusion test in continuous cell culture over 144 h, it was clearly shown that the synergistic interaction manifested itself not only as a potentiated cytostatic effect, but also as an increased cytotoxic activity, which was time-dependent (Figure 1).

Figure 2 shows cell cyle distribution of A-549 cells treated with ONC alone, lactone form of LVT alone, and the combination of both drugs. ONC alone (Figure 2b) at 1 $\mu g\ ml^{-1}$ slightly increased the proportion of cells in $G_2$/M phase, LVT alone at 5μM significantly decreased the proportion of cells in S phase from 33% to 13%, but had no effect at lower (2.5μM) concentration. Combination of both drugs (Figures 2f and 2g) resulted in a lowering of the proportion of S phase cells but to a lesser degree compared to 5μM LVT alone. The proportion of $G_1$ cells, however, is higher in the cultures treated with ONC + LVT than in the absence of these drugs. When these data are compared to growth curves, the latter indicating a very significant (several-fold) slow-down of cell proliferation, it is evident that the suppression of cell growth by combination of ONC and LVT is a result of the prolongation of the overall cell cycle, rather than the specific arrest in particular phase of the cycle. $G_1$ phase, however, is more

**Table IV** Clonogenicity of ASPC-1 and A-549 cells treated with ONCONASE and lovastatin, alone and in combination

| *Treatment group* | *Mean numbr of colonies formed (+/− S.D.)* ASPC-1 | A-549 |
|---|---|---|
| CTL | 57 (4.9) | 52 (4.2) |
| LVT 7.5 μM alone | 23 (7.1) | 9 (1.4)[a] |
| ONC 0.2 $\mu g\ ml^{-1}$ alone | 51 (4.2) | 38 (7.8) |
| ONC 2.5 $\mu g\ ml^{-1}$ alone | 31 (6.4) | 28 (12.0) |
| ONC 5.0 $\mu g\ ml^{-1}$ alone | 21 (0.7) | 10 (3.5) |
| LVT 7.5 μM + ONC 0.2 $\mu g\ ml^{-1}$ | 25 (3.5) | 0 (0)[a] |
| LVT 7.5 μM + ONC 2.5 $\mu g\ ml^{-1}$ | 7 (2.1) | 0 (0)[a] |
| LVT 7.5 μM + ONC 5.0 $\mu g\ ml^{-1}$ | 3 (1.4) | 0 (0)[a] |

S.D. = standard deviations; CTL = untreated controls, ASPC-1 1,000 cells plated/dish, and A-549 100 cells plated/dish, under the conditions specified in the Materials and methods; LVT = lovastatin lactone; ONC = ONCONASE. [a]In the A-549 cell system, lovastatin was also used at the 5 μM concentration, i.e., the same concentration as used in both the trypan blue dye exclusion and flow cytometry studies, and the mean numbers of colonies (with standard deviations in parentheses) were vertically 21 (3.5) for LVT alone, and 0 (0) for all combinations of LVT 5 μM with varying concentrations of ONC.

**Figure 1** Trypan blue dye exclusion cell viability assessments in the two human tumour cell lines, expressed as: **a**, the natural logarithm of an absolute number of viable ASPC-1 cells (abscissa) in time (ordinate); **b**, a time-dependent percentage of dead cells in the ASPC-1 cell system; **c**, the natural logarithm of an absolute number of viable A-549 cells (abscissa) in time (ordinate), and **d**, a time-dependent percentage of dead cells in the A-549 cell system. Symbols: O–O–O represents untreated controls; ●–●–● represents ONCONASE at 1 $\mu g\ ml^{-1}$ alone; □–□–□ represents Lovastatin at 7.5 μM concentration in ASPC-1 cells, and at 5 μM in A-549 cells, alone Δ–Δ–Δ represents a combination of both agents at the same concentrations, respectively. All cells were treated as described in the Material and methods section. The plotted points represent means of three values with standard deviations.

**Figure 2** DNA frequency distribution histograms of A-549 cells untreated and treated with ONC and LVT: **a**, untreated controls; **b**, cells treated with ONC alone at final concentration of 1 $\mu g\,ml^{-1}$ for 72 h; **c**, cells treated with ONC alone at final concentration of 0.5 $\mu g\,ml^{-1}$ for 72 h; **d**, cells treated with 5 $\mu M$ LVT alone for 72 h; **e**, cells treated with 2.5 $\mu M$ LVT alone for 72 h; **f**, cells treated with the combination of ONC at 1 $\mu g\,ml^{-1}$ and 5 $\mu M$ LVT for 72 h; **g**, cells treated with the combination of ONC at 0.5 $\mu g\,ml^{-1}$ and 2.5 $\mu M$ LVT for 72 h. Percentages of cells in different phases of the cell cycle are given in each panel.

prolonged than the remaining portion of the cycle. This is in contrast to the LVT alone at 5$\mu M$ which arrests cells quite specifically in $G_1$ phase.

While the cytostatic effects of the combined treatment with ONC and LVT are manifested early during the treatment (no cell death but significant inhibition of cell growth were seen during the first 72 h of treatment with 1 $\mu g\,ml^{-1}$ of ONC + 5 or 7.5$\mu M$ of LVT), the cytotoxic effects became apparent, and progressively increased, later (Figure 1).

To confirm the primary mechanism of action of LVT, HT-520 cells were subjected to both the acid and the lactone forms of LVT, with and without concomitant 200$\mu M$ mevalonic acid lactone (MVA) (Jakobisiak *et al.*, 1991). MVA partially reversed both the LVT-induced cell growth inhibition and the interactive capability of LVT; these results are presented in Table V and expressed as $ED_{50}$ values for the ONC + LVT combination in the MTT assay.

**Table V** HT-520 cell growth inhibition induced by ONCONASE alone or in combination with lovastatin and the reversal of inhibition by mevalonate, expressed as $ED_{50}$ values in MTT assay with standard deviations

| *Treatment group* | $ED_{50}$ | *S.D.* |
|---|---|---|
| ONC alone | 1.143 | (0.16) |
| ONC + 200 $\mu M$ MVA | 0.632 | (0.16) |
| ONC + 15aLVT | 0.001 | (0.00) |
| ONC + 15LVT | 0.054 | (0.01) |
| ONC + 15aLVT + 200 $\mu M$ MVA | 0.233 | (0.07) |
| ONC + 15LVT + 200 $\mu M$ MVA | 0.221 | (0.03) |

ONC = ONCONASE; MVA = mevalonate; 15aLVT = 15 $\mu M$ *in vitro* activated lovastatin, i.e., beta-hydroxy acid form; 15LVT = 15 $\mu M$ lovastatin lactone. The 15 $\mu M$ LVT lactone alone caused a mean 53.5% inhibition of tumour cell growth, which was decreased to a mean 19.8% at 200 $\mu M$ MVA. The 15 $\mu M$ aLVT alone caused a mean 69.5% inhibition of tumour cell growth, which was decreased to a mean 24.1% at 200 $\mu M$ MVA. 200 $\mu M$ MVA alone caused 9.9% inhibition of cell growth. Therefore, 200 $\mu M$ MVA reversed the cell growth inhibition induced by LVT lactone by 62.9%, and that induced by aLVT by 65%. In fact, since 200 $\mu M$ MVA alone was inhibitory to the cell growth, as also reflected by $ED_{50}$ value for ONC + MVA combination being lower than that of ONC alone, the actual reversals of cell growth inhibition have been greater than was actually observed.

### Discussion

Our present results show that the combinations of ONC with both acid and lactone forms of LVT demonstrate a synergistic anti-proliferative activity, as measured by the MTT colorimetric assay, clonogenicity studies and the trypan blue dye exclusion assessments. The flow cytometric studies indicated that the cytostatic effect induced by treatment with LVT + ONC results predominantly from the extension of duration of all phases of the cell cycle. $G_1$ phase, however, appears to be more prolonged than S and $G_2$/M by ONC + LVT combination, compared to the untreated cells. Interstingly, whereas LVT alone at 5$\mu M$ concentration produced the $G_1$ cell arrest, the addition of ONC partially abolished the $G_1$-specific effect of LVT, presumably by entrapping the cells in other phases of the cell cycle.

We have also demonstrated that these activites of LVT alone and in combination are reversible by MVA, thus confirming a primary mechanism of action of LVT as an inhibitor of HMG-CoA reductase. The findings of at least equal degree of anti-tumour activitiy exerted by LVT lactone suggest that this form of LVT can be activated by tumour cells, and that the *in vitro* activation of LVT lactone may not always be necessary. These findings have very important practical applications with regard to the potential use of LVT in *in vivo* systemic cancer treatment.

The ability of MVA, but not cholesterol, to reverse the HMG-CoA reductase inhibitor-induced cell growth arrest and DNA synthesis inhibition (Quesney-Huneeus *et al.*, 1979), as well as a direct stimulation of DNA synthesis in mouse fibroblasts upon microinjection of recombinant p21 *ras* proteins (Stacey & Kung, 1984), and the $G_1$ phase arrest of growing cells and the reversal of a transformed phenotype induced by microinjection of monoclonal anti-p21 antibodies (Kung *et al.*, 1986), all strongly suggest an essential cell growth regulatory activity of the inner plasma membrane-anchored p21 *ras* proteins. However, since LVT could interfere with the function of over 40 other proteins known to be normally isoprenylated (Madaule & Axel, 1985; Chardin & Tavitian, 1986; Touchot *et al.*, 1987; Casey *et al.*, 1989; Farnsworth *et al.*, 1989; Schmidt *et al.*, 1984), it is also possible that the observed anti-proliferative activity of this drug may be as well related to the interference with the function of other than, or in addition to, p21 *ras* protein(s), e.g., nuclear scaffold lamins (Farnsworth *et al.*, 1989; Vorburger *et al.*, 1989). These latter proteins undergo cell-cyclical proteolytic degradation which is associated with a generation of a 46 kD protein possessing a nucleoside triphosphatase activity. This activity, in turn, is thought to participate in nucleocytoplasmic transport of RNA (Tokes & Clawson, 1989). It has been previously demonstrated that, by analogy to the factor **a** of *Saccharomyces cerevisiae* and p21 *ras* proteins, the carboxyl-terminal cysteine of lamin B is farnesylated and its carboxyl group methylated (Anderegg *et al.*, 1988; Farnsworth *et al.*, 1989) and, interestingly, in a cell cycle-dependent manner (Chelsky *et al.*, 1987). All of these findings emphasise our lack of knowledge of a precise mechanism of action of LVT.

The observed synergism between ONC and LVT might be related to their effects on RNA metabolism: ONC through its ribonucleotyic activity (Ardelt *et al.*, 1991) capable of destroying specific species of RNA, and LVT acting via interference with the lamin proteins-related nucleocytoplasmic RNA transport (Tokes & Clawson, 1989). Both actions could conceivably inhibit cell growth.

An increased expression of a mutated ('activated') *ras* gene product(s) has been observed in a variety of human malignancies, including adenocarcinoma of the lung (Rodenhuis *et al.*, 1987), pre-malignant and high grade lesions of bladder carcinoma (Viola *et al.*, 1985), colon carcinoma (Forrester *et al.*, 1987), acute myeloid leukaemia (Bos *et al.*, 1985), ovarian serous cystadenocarcinoma (Feig *et al.*, 1984), and melanoma (Albino *et al.*, 1984). Using monoclonal antibodies specific for synthetic eight residues peptide containing amino acids corresponding to positions 10–17 of the mutated in positon 12 Ha-*ras* gene product (valine substituted for glycine), it was shown that mutated *ras* gene expression is markedly increased in most of the human colon and mammary carcinomas, but not in normal colonic and mammary epithelia, nor benign fibroadenoma and/or fibrocystic disease (Hand *et al.*, 1984).

Although an increased expression of normal (not mutated) human Ha-*ras* proto-oncogene has been shown to be capable of inducing tumourigenic transformation of mammalian cells (Chang *et al.*, 1982), such an enhanced expression appears to be infrequent in human neoplasia, with an approximate incidence of 1% (Barbacid, 1987), and in some instances it may only induce immortalisation of cells (Spandidos & Wilkie, 1984).

In majority of human tumours, the 'activation' (mutation) of *ras* genes did not seem to correlate with the histopathological properties of the tumour, and was not associated with any specific type of neoplasia (Barbacid, 1987; Forrester *et al.*, 1987). The 'activated' *ras* genes were detectable ony in some, but not other, tumour deposits of human metastatic melanoma isolated from the same patient, thus reflecting a significant tumour cell heterogeneity with regard to *ras* genes' expression (Albino *et al.*, 1984). Although the activated *ras* genes may not be able to induce malignant transformation by themselves, they can be effective as co-inducers of such transformation in cooperation with, e.g., nuclear oncogenes such as *c-myc* (Land *et al.*, 1983; Weinberg, 1989). All of these findings point to the still poorly understood relative clinical importance of both normally expressed and mutated *ras* gene products.

The observed synergism between ONC (currently being evaluated in Phase II human clinical trials) and LVT (which has been used in the treatment of certain forms of hypercholesterolemia), as reflected by both the increased cytostatic and cytotoxic effects, suggests that this combination should be investigated *in vivo*, including human trials. It may offer a new therapeutic approach against notoriously resistant human solid tumours.

The authors would like to thank Najma Khalid for excellent help in preparing the figures and tables.

Z.D. was supported in part by NCI grant R37 CA23296.

### References

ALBINO, A.P., LESTRANGE, R., OLIFF, A.I., FURTH, M.E. & OLD, L.J. (1984). Transforming *ras* genes from human melanoma: a manifestation of tumour heterogeneity? *Nature*, **308**, 69–72.

ANDEREGG, R.J., BETZ, R., CARR, S.A., CRABB, J.W. & DUNTZE, W. (1988). Structure of *Saccharomyces cerevisiae* mating hormone a-factor. Identification of S-farnesyl cysteine as a structural component. *J. Biol. Chem.*, **263**, 18236–18240.

ARDELT, W., MIKULSKI, S.M. & SHOGEN, K. (1991). Amino acid sequence of an anti-tumor protein from *Rana pipiens* oocytes and early embryos. Homology to pancreatic ribonucleases. *J. Biol. Chem.*, **266**, 245–251.

BARBACID, M. (1987). *ras* genes. *Ann. Rev. Biochem.*, **56**, 779–827.

BERENBAUM, M.C. (1981). Criteria for analysing interactions between biologically active agents. *Adv. Cancer Res.*, **35**, 269–335.

BOS, J.L., TOKSOZ, D., MARSHALL, C.J., VERLAAN-DE VRIES, M., VEENEMAN, G.H., VAN DER EB, A.J., VAN BOOM, J.H., JANSSEN, J.W.G. & STEENVOORDEN, A.C.M. (1985). Amino acid substitutions at codon 13 of the N-*ras* oncogene in human acute myeloid leukaemia. *Nature*, **315**, 726–730.

BRANDES, L.J., BOGDANOVIC, R.P., CAWKER, M.D. & LABELLA, F.S. (1987). Histamine and growth: interactions of antiestrogen binding site ligands with a novel histamine site that may be associated with calcium channels. *Cancer Res.*, **47**, 4025–4031.

BRUNO, S., CRISSMAN, H.A., BAUER, K.D. & DARZYNKIEWICZ, Z. (1991). Changes in cell nuclei during S phase: progressive chromatin condensation and altered expression of the proliferation-associated nuclear proteins Ki-67, cyclin (PCNA), p105, and p34. *Exp. Cell. Res.*, **196**, 99–106.

CASEY, P.J., SOLSKI, P.A., DER, C.J. & BUSS, J.E. (1989). p21ras is modified by a farnesyl isoprenoid. *Proc. Natl Acad. Sci. USA*, **86**, 8323–8327.

CHANG, E.H., FURTH, M.E., SCOLNICK, E.M. & LOWY, D.R. (1982). Tumorigenic transformation of mammalian cells induced by a normal human gene homologous to the oncogene of Harvey murine sarcoma virus. *Nature*, **297**, 479–483.

CHARDIN, P. & TAVITIAN, A. (1986). The *ral* gene: a new *ras* related gene isolated by the use of a synthetic probe. *EMBO J.*, **5**, 2203–2208.

CHELSKY, D., OLSON, J.F. & KOSHLAND, D.E. Jr (1987). Cell cycle-dependent methyl esterification of lamin B. *J. Biol. Chem.*, **262**, 4304–4309.

DARZYNKIEWICZ, Z., CARTER, S.P., MIKULSKI, S.M., ARDELT, W.J. & SHOGEN, K. (1988). Cytostatic and cytotoxic effects of Pannon (P-30 Protein), a novel anticancer agent. *Cell Tissue Kinet.*, **21**, 169–182.

DECLUE, J.E., VASS, W.C., PAPAGEORGE, A.G., LOWY, D.R. & WILLUMSEN, B.M. (1991). Inhibition of cell growth by lovastatin is independent of *ras* function. *Cancer Res.*, **51**, 712–717.

DOYLE, J.W. & KANDUTSCH, A.A. (1988). Requirement for mevalonate in cycling cells: quantitative and temporal aspects. *J. Cell. Physiol.*, **137**, 133–140.

FARNSWORTH, C.C., WOLDA, S.L., GELB, M.H. & GLOMSET, J.A. (1989). Human lamin B contains a farnesylated cysteine residue. *J. Biol. Chem.*, **264**, 20422–20429.

FEIG, L.A., BAST, R.C. Jr, KNAPP, R.C. & COOPER, G.M. (1984). Somatic activation of $ras^K$ gene in a human ovarian carcinoma. *Science*, **223**, 698–701.

FINEGOLD, A.A., SCHAFER, W.R., RINE, J., WHITEWAY, M. & TAMANOI, F. (1990). Common modifications of trimeric G proteins and *ras* protein: involvement of polyisoprenylation. *Science*, **249**, 165–169.

FORRESTER, K., ALMOGUERA, C., HAN, K., GRIZZLE, W.E. & PERUCHO, M. (1987). Detection of high incidence of K-*ras* oncogenes during human colon tumorigenesis. *Nature*, **327**, 298–303.

GILMAN, A.G. (1987). G proteins: transducers of receptor-generated signals. *Ann. Rev. Biochem.*, **56**, 615–649.

GULINO, A., BARRERA, G., VACCA, A., FARINA, A., FERRETTI, C., SCREPANTI, I., DIANZANI, M.U. & FRATI, L. (1986). Calmodulin antagonism and growth-inhibiting activity of triphenyl-ethylene antiestrogens in MCF-7 human breast cancer cells. *Cancer Res.*, **46**, 6274–6278.

HAND, P.H., THOR, A., WUNDERLICH, D., MURARO, R., CARUSO, A. & SCHLOM, J. (1984). Monoclonal antibodies of predefined specificity detect activated *ras* gene expression in human mammary and colon carcinomas. *Proc. Natl Acad. Sci. USA*, **81**, 5227–5231.

JAKOBISIAK, M., BRUNO, S., SKIERSKI, J. & DARZYNKIEWICZ, Z. (1991). Cell cycle-specific effects of lovastatin. *Proc. Natl Acad. Sci. USA*, **88**, 3628–3632.

KUNG, H.-F., SMITH, M.R., BEKESI, E., MANNE, V. & STACEY, D.W. (1986). Reversal of transformed phenotype by monoclonal antibodies against Ha-*ras* p21 proteins. *Exp. Cell Res.*, **162**, 363–371.

LAND, H., PARADA, L.F. & WEINBERG, R.A. (1983). Tumorigenic conversion of primary embryo fibroblasts requires at least two cooperating oncogenes. *Nature*, **304**, 596–602.

LOWE, D.G., CAPON, D.J., DELWART, E., SAKAGUCHI, A.Y., NAYLOR, S.L. & GOEDDEL, D.V. (1987). Structure of the human and murine R-ras genes, novel genes closely related to *ras* proto-oncogenes. *Cell*, **48**, 137–146.

MADAULE, P. & AXEL, R. (1985). A novel *ras*-related gene family. *Cell*, **41**, 31–40.

MIKULSKI, S.M., BERNSTEIN, E.H., ARDELT, W., SHOGEN, K. & MENDUKE, H. (1990*a*). Striking increase of survival of mice bearing M109 Madison carcinoma treated with a novel protein from amphibian embryos. *J. Natl Cancer Inst.*, **82**, 151–153.

MIKULSKI, S.M., VIERA, A., ARDELT, W., MENDUKE, H. & SHOGEN, K. (1990*b*). Tamoxifen and trifluoroperazine (Stelazine) potentiate cytostatic/cytotoxic effects of P-30 Protein, a novel protein possessing anti-tumour activity. *Cell Tissue Kinet.*, **23**, 237–246.

MINTZ, B. & ILLMENSEE, K. (1975). Normal genetically mosaic mice produced from malignant teratocarcinoma cells. *Proc. Natl Acad. Sci. USA*, **72**, 3585–3589.

MORI, T., TAKAI, Y., MINAKUCHI, R., YU, B. & NISHIZUKA, Y. (1980). Inhibitory action of chlorpromazine, dibucaine and other phospholipid-interacting drugs on calcium-activated, phospholipid-dependent protein kinase. *J. Biol. Chem.*, **255**, 8378–8380.

NEER, E.J. & CLAPHAM, D.E. (1988). Roles of G protein subunits in transmembrane signalling. *Nature*, **333**, 129–134.

PAPAIOANNOU, V.E., MCBURNEY, M.W., GARDNER, R.L. & EVANS, M.J. (1975). Fate of teratocarcinoma cells injected into early mouse embryos. *Nature*, **258**, 70–73.

PAPAIOANNOU, V.E. & ROSSANT, J. (1983). Effects of the embryonic environment on proliferation and differentiation of embryonal carcinoma cells. *Cancer Surv.*, **2**, 165–183.

QUESNEY-HUNEEUS, V., WILEY, M.H. & SIPERSTEIN, M.D. (1979). Essential role for mevalonate synthesis in DNA replication. *Proc. Natl Acad. Sci. USA*, **76**, 5056–5060.

REPKO, E.M. & MALTESE, W.A. (1989). Post-translational isoprenylation of cellular proteins is altered in response to mevalonate availability. *J. Biol. Chem.*, **264**, 9945–9952.

RODENHUIS, S., VAN DE WETERING, M.L., MOOI, W.J., EVERS, S.G., VAN ZANDWIJK, N. & BOS, J.L. (1987). Mutational activation of the K-*RAS* oncogene. A possible pathogenetic factor in adenocarcinoma of the lung. *N. Engl. J. Med.*, **317**, 929–935.

SCHAFER, W.R., KIM, R., STERNE, R., THORNER, J., KIM, S.-H. & RINE, J. (1989). Genetic and pharmacological suppression of oncogenic mutations in *RAS* genes of yeast and humans. *Science*, **245**, 379–385.

SCHMIDT, R.A., SCHNEIDER, C.J. & GLOMSET, J.A. (1984). Evidence of posttranslational incorporation of a product of mevalonic acid into Swiss 3T3 cell proteins. *J. Biol. Chem.*, **259**, 10175–10180.

SINENSKY, M. & LOGEL, J. (1985). Defective macromolecule biosynthesis and cell-cycle progression in a mammalian cell starved for mevalonate. *Proc. Natl Acad. Sci. USA*, **82**, 3257–3261.

SPANDIDOS, D.A. & WILKIE, N.M. (1984). Malignant transformation of early passage rodent cells by a single mutated human oncogene. *Nature*, **310**, 469–475.

STACEY, D.W. & KUNG, H.-F. (1984). Transformation of NIH 3T3 cells by microinjection of Ha-ras p21 protein. *Nature*, **310**, 508–511.

TOKES, Z.A. & CLAWSON, G.A. (1989). Proteolytic activity associated with the nuclear scaffold. The effect of self-digestion on lamins. *J. Biol. Chem.*, **264**, 15059–15065.

TOUCHOT, N., CHARDIN, P. & TAVITIAN, A. (1987). Four additional members of the *ras* gene superfamily isolated by an oligonucleotide strategy: molecular cloning of YPT-related cDNAs from a rat brain library. *Proc. Natl Acad. Sci. USA*, **84**, 8210–8214.

VIOLA, M.V., FROMOWITZ, F., ORAVEZ, S., DEB, S. & SCHLOM, J. (1985). *ras* oncogene p21 expression is increased in premalignant lesions and high grade bladder carcinoma. *J. Exp. Med.*, **161**, 1213–1218.

VORBURGER, K., KITTEN, G.T. & NIGG, E.A. (1989). Modification of nuclear lamin proteins by a mevalonic acid occurs in reticulocyte lysates and requires the cysteine residues of the C-terminal CXXM motif. *EMBO J.*, **8**, 4007–4014.

WEINBERG, R.A. (1989). Oncogenes, antioncogenes, and the molecular bases of multistep carcinogenesis. *Cancer Res.*, **49**, 3713–3721.